# Risk-adjusted antibiotic consumption in 34 public acute hospitals in Ireland, 2006 to 2014

**DOI:** 10.2807/1560-7917.ES.2016.21.32.30312

**Published:** 2016-08-11

**Authors:** Ajay Oza, Fionnuala Donohue, Howard Johnson, Robert Cunney

**Affiliations:** 1 Health Service Executive (HSE) Health Protection Surveillance Centre (HPSC), Dublin, Ireland; 2 Knowledge Management (incorporating Health Intelligence), Health Service Executive (HSE) Health and Wellbeing Directorate, Dublin, Ireland

**Keywords:** Antibiotic use, Benchmarking, Hospital Services, Patient Characteristics

## Abstract

As antibiotic consumption rates between hospitals can vary depending on the characteristics of the patients treated, risk-adjustment that compensates for the patient-based variation is required to assess the impact of any stewardship measures. The aim of this study was to investigate the usefulness of patient-based administrative data variables for adjusting aggregate hospital antibiotic consumption rates. Data on total inpatient antibiotics and six broad subclasses were sourced from 34 acute hospitals from 2006 to 2014. Aggregate annual patient administration data were divided into explanatory variables, including major diagnostic categories, for each hospital. Multivariable regression models were used to identify factors affecting antibiotic consumption. Coefficient of variation of the root mean squared errors (CV-RMSE) for the total antibiotic usage model was very good (11%), however, the value for two of the models was poor (> 30%). The overall inpatient antibiotic consumption increased from 82.5 defined daily doses (DDD)/100 bed-days used in 2006 to 89.2 DDD/100 bed-days used in 2014; the increase was not significant after risk-adjustment. During the same period, consumption of carbapenems increased significantly, while usage of fluoroquinolones decreased. In conclusion, patient-based administrative data variables are useful for adjusting hospital antibiotic consumption rates, although additional variables should also be employed.

## Introduction

Antibiotic consumption can vary between hospitals depending on a number of factors including implementation and adherence to antibiotic policies, antibiotic resistance rates, and hospital function which depends on the patient characteristics [1-3].

A few reports have focused on risk adjustment models that account for differences in specific health risks that patients bring to their healthcare facilities, thus ‘levelling the playing field’ when comparing rates of antibiotic consumption between hospitals with varied case mix [4-6]. These approaches provide a benchmarking tool to identify facilities that have consistently higher or lower than expected rates in order to encourage compliance with guidelines. As well as adopting these approaches to the Irish antibiotic consumption context, this study explored how changes in case mix over time can affect antibiotic usage.

Variables relating to a variety of patient characteristics from public acute hospitals, based on administrative data, are readily available in Ireland [7]. Unlike parameters for clinical services (such as provision of intensive care, oncology and cardiac services) which provide a static representation, patient-based parameters (age, sex, place of admission and discharge, diagnoses and procedures) can reflect changes in case mix over time.

The rates of antibiotic use in hospitals are dynamic and have been shown to change over time not only in Ireland but in many countries [8]. Hospital administrative data are therefore a good candidate for developing risk adjustment models. The aim of this study was to investigate the usefulness of patient-based administrative data variables for adjusting hospital antibiotic consumption rates.

## Methods

### Study design

The study was an observational, retrospective analysis of aggregate data on antibiotic use and patient administration from 34 public acute hospitals in Ireland. The participating hospitals in this study represented all tertiary/referral hospitals in Ireland and all general hospitals bar two facilities that were unable to provide consistent antibiotic consumption data. Single-speciality hospitals (maternity, paediatric or orthopaedic) were excluded.

### Data sources

Clinical antimicrobial dispensary data from hospital pharmacy systems were extracted and converted into defined daily doses (DDD) using the World Health Organization (WHO) Anatomical Therapeutic Chemical (ATC) classification method [9] via the MicroB secure online healthcare data analytical system [10]. Drugs dispensed to non-acute or non-inpatient areas were excluded. The rates for antibiotics were expressed as DDD per 100 bed-days used (BDU) and grouped into the following outcome variables:

1. Carbapenems, which included agents such as meropenem,

2. Fluoroquinolones such as ciprofloxacin,

3. Glycopeptides such as vancomycin and teicoplanin (excluding oral use),

4. Macrolides such as erythromycin,

5. Penicillins with enzyme inhibitors such as amoxicillin/clavulanic acid,

6. Third-generation cephalosporins such as cefotaxime,

7. Total antibiotic use, all systemic anti-bacterial agents.

Hospital In-patient Enquiry (HIPE) data following patients’ discharge or death in the hospital were used to obtain aggregate annual patient administration variables from 2006 to 2014 for all participating hospitals. These were accessed through the Health Intelligence Ireland secure online healthcare data analytical system [11]. Data on non-inpatients (day cases and outpatients) were excluded from the analysis.

### Statistical Analysis

R software was used for all statistical analyses [12]. We constructed log-normal regression models for each of the seven outcome variables using a stepwise forward selection method to identify risk and protective variables [13]. Collinear variables were removed following each selection. A categorical variable representing year was also entered.

Results for these models are reported using incidence rate ratios (coefficient estimate) and 95% confidence intervals for each outcome variable. Coefficients of variation of the root mean squared errors (CV-RMSE) are reported for each model.

Satisfactory models were used to generate expected values of antibiotic consumption for all facilities for each year in the study period, given the patient administration parameters for the facilities during the relevant time points. The difference between the observed antibiotic use and the estimated use is the residual, and the standardised residual is a ratio of the residual divided by the standard deviation of the residuals. Data points for any facility–year combination that had standardised residual values of less than − 2 or greater than + 2 were considered as having lower or higher than expected consumption, as estimated by the statistical model, respectively.

## Results

### Descriptive analysis

The total antibiotic usage rate for the 34 participating public acute hospitals decreased from 82.5 in 2006 to 80.0 DDD/100 BDU in 2009, and then increased to 89.2 DDD/100 BDU in 2014. Rates for carbapenems, glycopeptides and penicillins with enzyme inhibitors increased, those for macrolides and third-generation cephalosporins stayed level and those for fluoroquinolones decreased between 2006 and 2014 (Table 1).

**Table 1:** Consumption rates of five antibiotic groups and total antibiotics, Ireland, 2006–2014

Year	Carbapenems	Fluoroquinolones	Glycopeptides	Macrolides	Penicillins with enzyme inhibitors	Third-generation cephalosporins	Total antibiotics
2006	1.2 (0.1–4.9)	10.3 (5.0–30.0)	2.4 (0.2–7.0)	12.3 (5.4–20.3)	20.5 (14.5–36.5)	1.9 (0.3–3.7)	82.5 (56.6–118.1)
2007	1.2 (0.0–3.0)	10.2 (6.2–27.8)	2.3 (0.4–4.7)	11.7 (6.3–20.5)	21.4 (13.3–38.7)	1.6 (0.6–3.2)	80.6 (61.6–105.8)
2008	1.9 (0.1–6.2)	8.7 (5.3–28.1)	2.6 (0.3–6.5)	11.8 (6.5–20.3)	22.1 (11.6–40.9)	1.6 (0.3–3.2)	81.9 (58.1–116.2)
2009	2.3 (0.1–6.7)	6.5 (0.6–26.2)	2.9 (0.2–7.2)	11.0 (5.8–20.2)	22.6 (14.6–39.4)	1.5 (0.4–3.0)	80.0 (63.3–112.8)
2010	2.6 (0.4–7.9)	6.1 (1.6–11.7)	3.0 (0.4–7.8)	11.4 (5.6–21.8)	24.0 (14.3–38.9)	1.6 (0.4–3.7)	83.4 (63.0–124.9)
2011	2.6 (0.2–7.5)	6.2 (2.5–12.1)	3.2 (0.6–8.0)	12.4 (5.9–23.2)	26.2 (18.2–42.5)	1.6 (0.7–3.4)	87.9 (67.0–135.6)
2012	3.0 (0.5–9.4)	6.3 (2.7–12.5)	3.0 (0.4–5.2)	12.7 (5.9–27.9)	27.4 (19.7–42.9)	1.7 (0.2–4.0)	88.6 (66.6–126.7)
2013	3.7 (0.2–9.6)	5.9 (2.5–11.0)	3.2 (0.5–5.4)	12.2 (4.8–25.5)	27.0 (19.7–40.6)	1.6 (0.1–3.8)	87.3 (62.3–114.8)
2014	4.1 (0.5–9.0)	5.9 (2.6–11.5)	3.5 (0.6–5.7)	12.2 (3.3–21.4)	26.9 (16.1–40.9)	1.8 (0.2–4.3)	89.2 (45.7–129.1)

Rates are given in defined daily doses per 100 bed-days used, with minimum-to-maximum range in parentheses.

**Figure 1:**
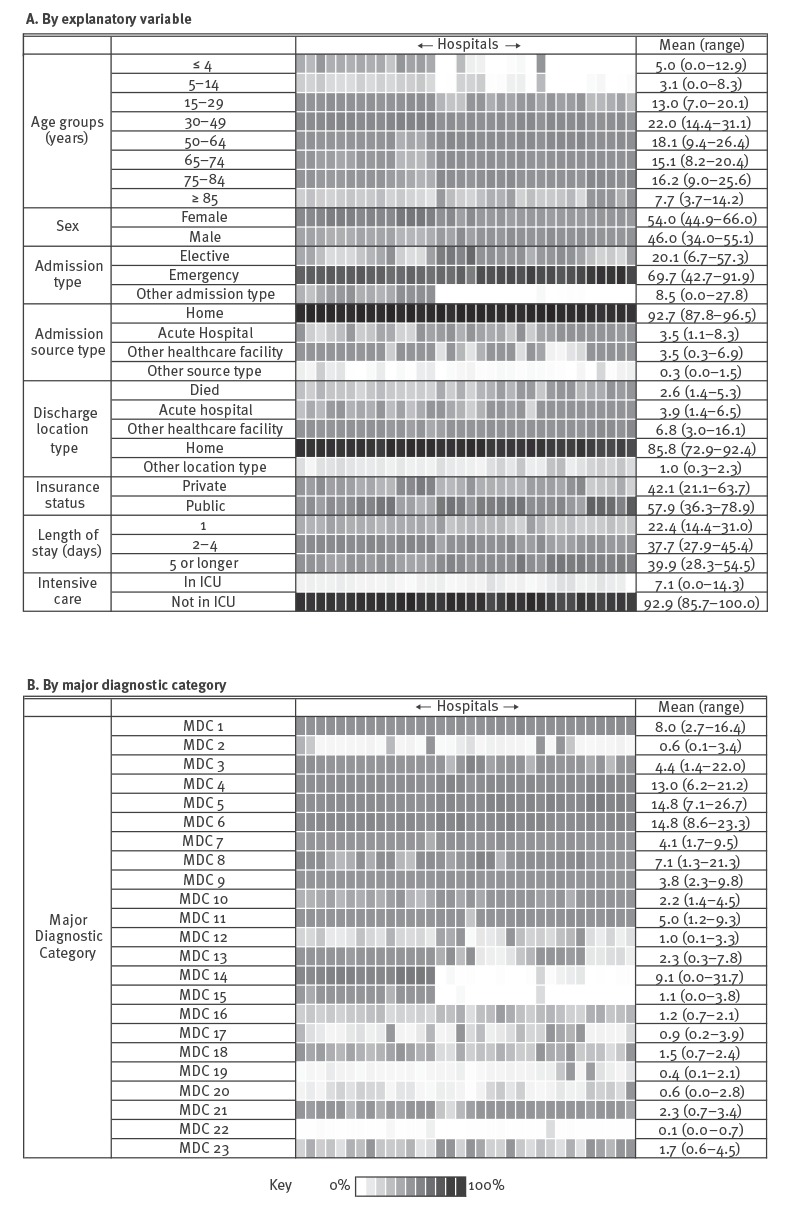
Heat map showing percentage of 34 administration variables for antibiotic consumption in public acute hospitals, Ireland, 2006–2014

ICU: intensive care unit; MDC: major diagnostic categories.

A heat map of the 29 explanatory variables grouped into eight sections is shown in Figure 1A and the 23 major diagnostic categories (MDC) in Figure 1B [14]. Each section for each hospital represents 100% of all discharged patients over the entire study period. Note that while the figures show combined values for all nine years for each hospital, individual data points for each year, hospital and explanatory variable were used in the regression analysis.

The variables are averages over the study period. For panel A, they are grouped into eight sections which each total 100% for any hospital.

MDC 1: diseases and disorders of the nervous system; MDC 2: diseases and disorders of the eye; MDC 3: diseases and disorders of the ear, nose, mouth and throat; MDC 4: diseases and disorders of the respiratory system; MDC 5: diseases and disorders of the circulatory system; MDC 6: diseases and disorders of the digestive system; MDC 7: diseases and disorders of the hepatobiliary system and pancreas; MDC 8: diseases and disorders of the musculoskeletal system and connective tissue; MDC 9: diseases and disorders of the skin, subcutaneous tissue and breast; MDC 10: endocrine, nutritional and metabolic diseases and disorders; MDC 11: diseases and disorders of the kidney and urinary tract; MDC 12: diseases and disorders of the male reproductive system; MDC 13: diseases and disorders of the female reproductive system; MDC 14: pregnancy, childbirth and the puerperium; MDC 15: newborns and other neonates; MDC 16: diseases and disorders of the blood and blood forming organs and immunological disorders; MDC 17: neoplastic disorders (haematological and solid neoplasms); MDC 18: infectious and parasitic diseases; MDC 19: mental diseases and disorders; MDC 20: alcohol/drug use and alcohol/drug-induced organic mental disorders; MDC 21: injuries, poisoning and toxic effects of drugs; MDC 22: burns; MDC 23: factors influencing health status and other contacts with health services.

The proportion of patients aged 30 to 49 years ranged from 14% to 31% between hospitals and accounted for the highest proportions of all discharged patients, i.e. 22% of the all patients. The age group five to 14 years represented the lowest proportion of patients. The proportion of female patients ranged from 45% to 66% between hospitals and overall, 54% of all patients were female.

The most common type of admission was emergency, representing 70% of all discharges. Note that other admission types includes newborn and maternity admission. Among the admission sources, home was the most common type (88–97%), while ‘other source type’ (prison, psychiatric unit or temporary residence) was the least common at 0–2%. Among discharge locations, home was again the most common type (73–92%), while ‘other location type’ (prison, psychiatric unit or rehabilitation facility) was the least common at 0–2%. Just over half of the patients (58%) did not have private health insurance and the proportion of patients under a public payment scheme ranged from 36% to 79% per hospital.

Length of stay of one day (overnight) ranged from 14% to 31%, while a larger proportion stayed for between two to four days (28–45%); the remainder stayed five days or longer (28–55%). Overall, 7% of the patients had a stay in intensive care. Diseases and disorders of the circulatory system (MDC-5) and diseases and disorders of the digestive system (MDC-6) were the most common MDC overall at 15% each. Diseases and disorders of the respiratory system (MDC-4) were also frequent at 13%. Of interest for antibiotic consumption are infectious and parasitic diseases (MDC-18) which was uncommon at just over 1%.

### Regression analysis

The final seven regression models for the antimicrobial groups are shown in Table 2. Note that only the variables that had a statistically significant association with any of the antimicrobial groups are shown. The model performance indicator, CV-RMSE, for total antibiotic use was only 11% indicating this to be a very good model. However, the CV-RMSE for fluoroquinolones was 36% and 31% for third-generation cephalosporins, indicating these to be poor models. The remaining models were adequate.

**Table 2:** Incidence rate ratios of antibiotic consumption in public acute hospitals with 95% confidence intervals for the final seven regression models with percent CV-RMSE values, Ireland, 2006–2014

Outcome variable	Significant variables		Incidence rate ratio (95% CI)	CV-RMSE
Carbapenem usage	Age groups (years)	≤ 4	1.09 (1.06–1.12)	21%
		5–14	0.94 (0.91–0.96)	
		75–84	0.96 (0.95–0.97)	
		≥ 85	0.83 (0.81–0.86)	
	Admission source type	Acute hospital	0.54 (0.45–0.64)	
		Other source type	1.10 (1.06–1.13)	
	Discharge location type	Other location type	1.01 (1.01–1.02)	
	Intensive care	In ICU	1.02 (1.00–1.03)	
	Major diagnostic category	MDC 3	0.92 (0.90–0.94)	
		MDC 5	0.98 (0.96–1.00)	
		MDC 6	0.94 (0.92–0.95)	
		MDC 9	1.09 (1.05–1.14)	
		MDC 10	0.72 (0.65–0.80)	
		MDC 11	1.11 (1.08–1.15)	
		MDC 22	1.44 (1.12–1.86)	
	Year	2008	1.46 (1.16–1.85)	
		2009	1.77 (1.41–2.23)	
		2010	1.94 (1.55–2.43)	
		2011	1.95 (1.56–2.45)	
		2012	2.20 (1.76–2.76)	
		2013	2.35 (1.87–2.95)	
		2014	2.44 (1.94–3.07)	
Fluoroquinolone usage	Age group (years)	≥ 85	0.97 (0.94–1.00)	36%
	Admission source type	Acute Hospital	0.72 (0.64–0.82)	
	Length of stay (days)	1	0.98 (0.97–0.99)	
	Intensive care	In ICU	1.02 (1.01–1.04)	
	Major diagnostic category	MDC 5	1.06 (1.04–1.08)	
		MDC 6	1.06 (1.04–1.08)	
		MDC 7	1.07 (1.03–1.12)	
		MDC 8	1.03 (1.02–1.04)	
		MDC 9	1.03 (1.00–1.06)	
		MDC 14	1.03 (1.02–1.04)	
		MDC 18	1.12 (1.03–1.22)	
		MDC 19	1.09 (1.05–1.12)	
		MDC 20	1.16 (1.07–1.26)	
		MDC 22	0.68 (0.48–0.97)	
	Year	2009	0.72 (0.62–0.85)	
		2010	0.61 (0.51–0.73)	
		2011	0.67 (0.56–0.80)	
		2012	0.75 (0.62–0.90)	
		2013	0.75 (0.62–0.91)	
		2014	0.73 (0.60–0.89)	
Glycopeptide usage	Age group (years)	50–64	1.02 (1.01–1.03)	23%
	Admission source type	Acute Hospital	0.90 (0.77–1.05)	
		Other healthcare facility	1.16 (1.09–1.24)	
		Other source type	1.08 (1.04–1.11)	
	Discharge location type	Acute hospital	0.95 (0.93–0.96)	
		Other healthcare facility	0.88 (0.82–0.95)	
	Length of stay (days)	1	1.03 (1.02–1.04)	
	Intensive care	In ICU	1.03 (1.01–1.04)	
	Major diagnostic category	MDC 2	1.04 (1.01–1.08)	
		MDC 6	0.98 (0.96–0.99)	
		MDC 10	0.93 (0.87–0.99)	
		MDC 11	1.10 (1.07–1.14)	
		MDC 12	0.92 (0.85–1.00)	
		MDC 16	0.71 (0.61–0.82)	
		MDC 17	1.32 (1.24–1.40)	
		MDC 21	0.89 (0.83–0.95)	
		MDC 22	1.71 (1.29–2.26)	
Macrolide usage	Age groups (years)	50–64	1.02 (1.02–1.03)	20%
		75–84	0.99 (0.98–0.99)	
	Admission source type	Acute hospital	0.88 (0.83–0.95)	
	Discharge location type	Other healthcare facility	0.94 (0.89–1.00)	
	Major diagnostic category	MDC 2	0.93 (0.90–0.96)	
		MDC 3	0.99 (0.98–1.00)	
		MDC 4	1.03 (1.02–1.04)	
		MDC 5	0.99 (0.99–1.00)	
		MDC 9	0.96 (0.94–0.98)	
		MDC 11	0.96 (0.95–0.98)	
		MDC 17	0.88 (0.85–0.91)	
	Year	2009	0.84 (0.76–0.93)	
		2010	0.90 (0.81–0.99)	
Usage of penicillins with enzyme inhibitor	Admission source type	Other healthcare facility	0.95 (0.92–0.98)	19%
	Discharge location type	Other location type	0.99 (0.99–1.00)	
	Length of stay (days)	2–4	0.99 (0.99–1.00)	
	Major diagnostic category	MDC 2	0.93 (0.91–0.96)	
		MDC 3	1.01 (1.00–1.01)	
		MDC 4	1.02 (1.01–1.03)	
		MDC 9	0.98 (0.96–1.00)	
		MDC 18	1.05 (1.00–1.10)	
Third-generation cephalosporin usage	Age group (years)	≤ 4	1.03 (1.00–1.06)	31%
		5–14	1.03 (1.01–1.05)	
	Admission source type	Other source type	1.10 (1.06–1.14)	
	Discharge location type	Other healthcare facility	0.80 (0.73–0.88)	
		Other location type	1.00 (1.00–1.01)	
	Length of stay (days)	1	0.97 (0.96–0.98)	
	Major diagnostic category	MDC 1	1.06 (1.04–1.08)	
		MDC 3	0.97 (0.95–0.98)	
		MDC 7	1.06 (1.03–1.10)	
		MDC 10	0.87 (0.79–0.95)	
		MDC 13	1.11 (1.08–1.14)	
		MDC 18	1.28 (1.18–1.40)	
		MDC 22	0.42 (0.29–0.62)	
Total antibiotic usage	Age group (years)	65–74	1.01 (1.00–1.01)	11%
	Admission source type	Acute hospital	0.91 (0.87–0.95)	
		Other source type	1.03 (1.02–1.04)	
	Discharge location type	Other healthcare facility	0.94 (0.91–0.97)	
	Intensive care	In ICU	1.01 (1.01–1.01)	
	Major diagnostic category	MDC 1	0.99 (0.98–0.99)	
		MDC 2	0.98 (0.97–1.00)	
		MDC 3	0.99 (0.99–0.99)	
		MDC 7	1.02 (1.01–1.03)	
		MDC 8	1.00 (1.00–1.01)	
		MDC 10	0.97 (0.95–1.00)	
		MDC 17	1.02 (1.00–1.04)	
		MDC 18	1.04 (1.01–1.08)	
		MDC 20	1.08 (1.05–1.12)	
		MDC 22	0.82 (0.73–0.93)	
	Year	2009	0.93 (0.87–0.99)	

CI: confidence interval; CV-RMSE: coefficients of variation of the root mean squared errors; ICU: intensive care unit; MDC: major diagnostic category.

Only the variables that had a statistically significant association with any of the antimicrobial groups are shown.

For the list of diseases in the different MDC, see Figure 1.

Different age groups were associated with increased risk of consumption of the different antibiotic groups. In particular, there was a high degree of association between decreased use of carbapenem and hospitals with a higher proportion of patients in the age group of five to 14 year-olds. Female sex was not significantly associated with any of the indicators of consumption, and neither was admission type (emergency, elective or other admission types).

Hospitals that had a higher proportion of patients admitted from ‘other source type’ had much reduced consumption of carbapenems, fluoroquinolones, glycopeptides, macrolides and total antibiotics. Similarly, hospitals that had a higher proportion of patients discharged to ‘other location type’ had much reduced consumption of glycopeptides, macrolides, third-generation cephalosporins and total antibiotics.

While only 0.1% of patients overall were classed under the MDC for burns (MDC 22), the category was associated with increased use of carbapenems and glycopeptides and with reduced use of fluoroquinolones and third-generation cephalosporins. The group of infectious and parasitic diseases (MDC 18) was associated with increased use of fluoroquinolones, penicillins with enzyme inhibitors, third-generation cephalosporins and total antibiotics.

Year as a categorical variable was significant for consumption of two antibiotic groups: carbapenems, which increased, and fluoroquinolones, which decreased over the study period. Two individual year values for macrolides (2009 and 2010) and one for total use (2009) were significant decreases.

### Outliers

**Figure 2:**
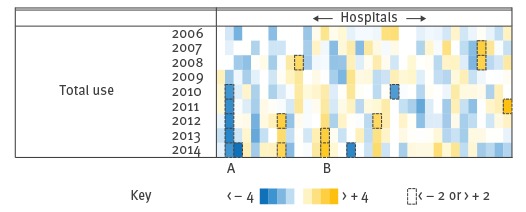
Heat map showing the variation in standardised residual for total antibiotic consumption in 34 public acute hospitals, Ireland, 2006–2014

Figure 2 shows the variation in standardised residuals. Each data point with a standardised residual greater than + 2 represented a time period of overuse of antibiotics at a particular hospital that was significantly greater than would be expected given the individual hospital’s patient profile. Similarly, standardised residuals lower than − 2 represented periods of significant underuse. For example, the hospital labelled A exhibited a reduction in consumption larger than expected for the patient profile of that hospital, and conversely, the hospital labelled B showed an overall increase. While this method of visualisation can be applied to subclasses of antibiotics, only the data from the model for total use is shown in Figure 2 as this model had the best model performance indicator, a CV-RMSE of 11%.

## Discussion

Our analysis identified three aspects of surveillance of hospital antimicrobial consumption: it identified factors that are important in driving antimicrobial use; it identified antibiotic groups for which the changes in consumption rate occur faster than could be explained by changes in patient profiles at the individual hospital alone; and it identified outliers so that stewardship strategies can be followed at those facilities to improve patient care.

On the first aspect, the main patient profile factors including MDC, and their variation both over time and across different facilities, may explain the dynamics that are evident in hospital antimicrobial consumption in Ireland. The range of factors that were significant for the different antimicrobial groups shows that using only a single factor such as the cost-based case mix index would not have been adequate [15]. Additional factors that could have been employed include clinical services parameters in conjunction with administration data or variables that define patient profiles at a finer level such as specific diagnosis/procedure codes [5,6].

On the second aspect, fluoroquinolones were the only antimicrobials in this study for which consumption decreased. There has been a concerted effort since 2008 by pharmacists in Ireland to reduce fluoroquinolone use as a whole and to switch to oral preparations as fluoroquinolones have a good bioavailability [16]. The increase in carbapenems is a concern as carbapenem-resistant *Enterobacteriaceae* are becoming more frequent across Europe [17]. All hospitals and the health service in Ireland have a collective responsibility to ensure that these increases are curtailed.

On the last aspect of outliers, our analysis showed that there were hospitals that had consistently higher antibiotic consumption than would be expected given the characteristics of patients cared for in those hospitals. It is likely that these few hospitals have services that were not included in the parameters of our models. It is important to address the presence of and adherence to antibiotic prescribing policies in these institutions.

Our analysis has limitations. Firstly, there is a debate about how to appropriately measure antibiotics usage. We selected the WHO ATC/DDD system as it is the one chosen by the European Surveillance of Antimicrobial Consumption Network (ESAC-Net). Furthermore, direct measures of antibiotic usage such as days of therapy could not be used as the pharmacy computer systems used in Ireland do not yet support it, unlike hospitals elsewhere [18]. The second limitation is the choice of denominator to express rates of use, of which there are also different viewpoints in the literature such as using number of admissions or discharges, or bed-days (or patient-days) used [19]. We selected bed-days used, as this denominator takes into account the average length of stay. However, given the strong association between length of stay of one day and total antibiotic use, number of admissions may be a more appropriate denominator. The third limitation is the possible presence of coding errors, and although hospital administration data in Ireland are increasingly used for research purposes, further validation is warranted [7]. The fourth limitation is the choice of regression method. Again, a variety of approaches have been attempted in the literature, ranging from indirect/direct standardisation, Poisson and negative-binomial regression, to simple linear regression [15,18,20]. We selected log-normal regression as the data fitted this distribution and satisfied its assumptions. Generalised estimating equations or mixed effects models were not required as it was the aim of our study to show differences between hospitals and adjust them via the explanatory variables [21]. The choice of modelling method also allowed for the use of conventional methods of analysis rather than employing complex procedures to compensate for overdispersion. However, the CV-RMSE for two of the models were very large and use of additional explanatory parameters is warranted. The last limitation is the sample size of only 34 hospitals. Even after including private and single-speciality hospitals, the population base would remain the overriding limit for any study conducted in Ireland. Extending the methodology to include other countries would be the only way to overcome this limitation.

Based on the findings of this study we recommend that the national guidance documents for antimicrobial stewardship should be updated to strengthen prescribing practice for carbapenems in particular and to incorporate a mechanism to ensure good adhere to antibiotic prescribing. We also recommend that performance-linked measures are put in place to ensure that when hospitals demonstrate reduction in the use of one antibiotic group, this does not lead to increases in another group of antibiotics. However, high antibiotic use among outliers may not imply poor performance and the hospitals not found to be outliers may still have substantial inappropriate use. Therefore, the findings of this study should be used in conjunction with other information and as part of a broader stewardship strategy. Finally, we suggest that a Europe-wide hospital antimicrobial study based on a unified methodology of risk adjustment is undertaken that takes into account the limitations of this and other similar studies. Risk adjustment may even be required to compare the wide variation in hospital antibiotic consumption as driven by diverse healthcare delivered to the populations in different jurisdictions.

In conclusion, patient-based administrative data variables are useful for adjusting hospital antibiotic consumption rates, although additional variables relating to clinical services should also be employed.
